# Characteristics and treatment strategies of mitral regurgitation associated with undifferentiated papillary muscle

**DOI:** 10.1007/s11748-012-0055-x

**Published:** 2012-05-11

**Authors:** Ichiro Matsumaru, Koji Hashizume, Tsuneo Ariyoshi, Kenta Izumi, Daisuke Onohara, Shun Nakaji, Mizuki Sumi, Kiyoyuki Eishi, Akira Tsuneto, Tomayoshi Hayashi

**Affiliations:** 1 Department of Cardiovascular Surgery, Nagasaki University Hospital, 1-7-1 Sakamoto, Nagasaki, 852-8501 Japan; 2 Department of Cardiovascular Medicine, Graduate School of Biomedical Sciences, Nagasaki University, 1-7-1 Sakamoto, Nagasaki City, Nagasaki 852-8501 Japan; 3 Department of Pathology, Nagasaki University Hospital, 1-7-1 Sakamoto, Nagasaki City, Nagasaki 852-8501 Japan

**Keywords:** Papillary muscles, Chordae tendineae, Mitral valve insufficiency, Mitral valve plasty, Echocardiography

## Abstract

**Purpose:**

In this report we review our experience of operations on mitral regurgitation associated with abnormal papillary muscles/chordae tendineae of the mitral valves and discussed the clinical characteristics, operative findings, and treatment strategies.

**Methods:**

Undifferentiated papillary muscle was defined as a hypoplastic chordae tendineae with anomalous formation of papillary muscles attached to the mitral valves directly. Consecutive 87 patients undergoing surgery for mitral regurgitation at our institution were reviewed and 6 of them had undifferentiated papillary muscle.

**Results:**

The underlying mechanism of regurgitation was prolapse at the center of the anterior leaflet in 3 cases and tethering, a wide area of myxomatous degeneration, and annular dilatation in one case, respectively. Five patients underwent mitral valve plasty and 1 patient received replacement. Anomalous formation of chordae tendineae was corrected by resection and suture with transplantation at the tip of the leaflet to which abnormal chordae were attached in 2 cases, while resection and suture with chordal shortening was performed in 1 case, and chordal reconstruction using artificial chordae was employed in 2 cases. There was no operative death, and postoperative echocardiography showed no residual regurgitation in any of the cases.

**Conclusions:**

Mitral regurgitation associated with undifferentiated papillary muscle resulted from prolapse or tethering and impaired flexibility of leaflets. It was possible to successfully treat the patients by mitral valve plasty unless complex congenital cardiac malformation coexisted. Detailed examinations of attached papillary muscle by echocardiography and intraoperative inspection are necessary and surgical techniques should be selected appropriately in each case.

## Introduction

The abnormalities of papillary muscle/chordae tendineae of the mitral valves (MVs) are a relatively rare form of congenital mitral valve (CMV) dysplasia, and hypoplastic chordae tendineae with anomalous formation of papillary muscles are considered to be a subtype of asymmetric parachute-like MVs [1, 2]. However, little is known about their clinical characteristics and treatment. In this report we review our experience of operations on mitral regurgitation (MR) associated with abnormal papillary muscles/chordae tendineae and discussed the clinical characteristics, operative findings, and treatment strategies.

## Patients and methods

### Definition of undifferentiated papillary muscle

**Fig. 1:**
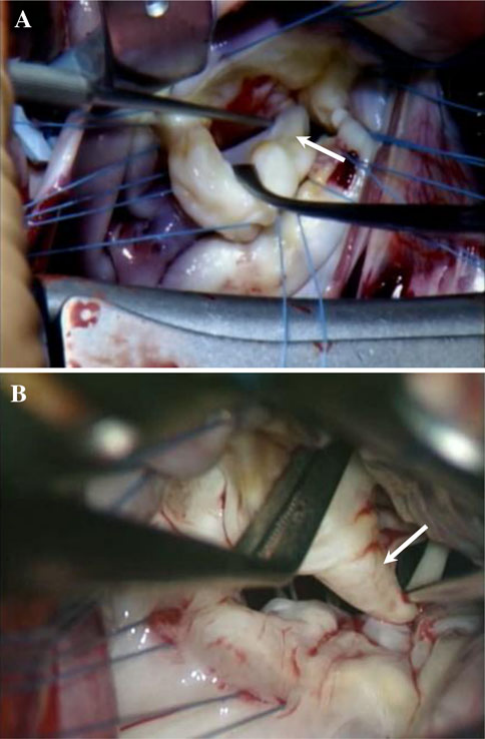
**a** Case 3, **b** case 4. Intra-operative findings of anomalies of mitral subvalvular apparatus Nerve hook hangs the club-like thickened papillary muscle which attached directly to the MVs with markedly poor development of chordae tendinae (*arrows*)

Undifferentiated papillary muscle was defined as a papillary muscle with club-like thickening, was often attached to the MVs directly due to a defect or marked underdevelopment of chordae tendineae that leads to compromised flexibility of papillary muscle/chordae tendineae/valve leaflet assembly during the cardiac contraction cycle (Fig 1a, b).

### Patients

Consecutive 87 patients undergoing surgery for MR at our institution from August 2008 to August 2010 were reviewed and 6 of them (6.9 %) had abnormalities in papillary muscle/chordae tendineae. The patients were 3 children (2 boys and 1 girl; average age ± SD, 14.0 ± 0.8 years) and 3 adults (3 males; average age, 62.3 ± 7.8 years). These cases were categorized preoperatively according to the New York Heart Association (NYHA) classification, as follows: class I, 2 cases; class II, 3 cases, and class III, 1 case. Electrocardiogram showed sinus rhythm in 4 patients and chronic atrial fibrillation in 2. The cardio-thoracic ratio on chest X-ray was 56.3 ± 6.2 % on average. Concomitant heart diseases in the 6 patients were: aortic valve insufficiency in 2, supra-valvular aortic stenosis in 1, tricuspid valve insufficiency in 1, and atrial septal defect (ASD; including patent foramen ovale) in 2 (Table 1).

**Table 1:** Clinical and demographic data of patients

Patient no.	Age	Sex	NYHA	ECG	CTR (%)
Case 1	13	M	III	SR	52
Case 2	14	F	II	SR	47
Case 3	15	M	II	SR	58
Case 4	53	M	I	Caf	66
Case 5	62	M	I	SR	54
Case 6	72	M	II	Caf	61

*NYHA* New York Heart Association, *ECG* electrocardiogram, CTR cardio-thoracic ratio, *SR* sinus rhythm,* Caf* chronic atrial fibrillation

### Echocardiography

Anatomical sites of MV leaflets were categorized according to Carpentier’s classification [3]. The degree of MR was assessed by the ratio of the maximum regurgitation area to the size of the left atrial, as follows: 0, none; 0.5, trivial; 1+, mild; 2+, moderate; 3+, moderate to severe; and 4+, very severe. In all cases, trans-thoracic echocardiography (TTE) was performed preoperatively, and intraoperative trans-esophageal echocardiography (TEE) was also performed by a cardiologist after induction of general anesthesia.

### Operative procedures

Surgery was carried out under cardiac arrest in 5 cases and under ventricular fibrillation through right thoracotomy in 1 case with moderate hypothermic cardiopulmonary bypass. MVs were exposed by making an incision in the right side of the left atrium and the leaflets, papillary muscle, and chordae tendineae were carefully inspected. Intraoperative videos were taken in all cases, and anatomical findings and operative procedures were recorded.

### Postoperative follow-up

Patients were followed at our institution and examined by echocardiography 6–12 months after surgery.

## Results

### Echocardiographic findings

Preoperative TTE showed that MR grade was moderate to severe in three cases and very severe, respectively. The mean left ventricular (LV) ejection fraction (EF) was 63.7 ± 6.7 %. The mean LV diastolic and systolic dimensions were 61.3 ± 7.0 and 40.2 ± 6.3 mm, respectively, with the mean left atrial dimension of 49.5 ± 10.8 mm. The underlying mechanism of regurgitation was prolapse at the center of the AML in 3 cases and tethering, a wide area of myxomatous degeneration, and annular dilatation in one case, respectively. Abnormality in papillary muscle-chordae tendineae was suspected in only one case by preoperative TTE and in 5 cases by intraoperative TEE.

### Operative findings

**Fig. 2:**
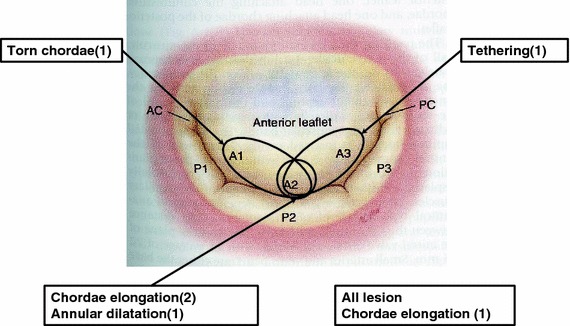
Location of anomalous PM and etiologies

Anomalous chordae tendineae were found at A_2_ in 3 cases, at A_1–2_ in 1 case, at A_2–3_ in 1 case, and in the whole area in 1 case. Associated valvular dysfunction was categorized according to Carpentier’s classification [3], into type 2 in 4 cases (chordae elongation, 3; torn chordae, 1), type 3 (tethering) in 1 case, and type 1 (annular dilatation) in 1 case (Fig. 2). Other operative findings were: P_2_ prolapse in 1 case; an accessory chorda in 1 case; and aortic valve hypoplasia in 1 case (Table 2).

**Table 2:** Preoperative trans-thoracic echocardiography findings

Patient no.	MR	Etiologies	EF (%)	Associated cardiac anomalies
Case 1	4+	A2 tethering	68	Supra-aortic valve stenosis
Case 2	3+	A2-3 prolapse	64	ASD accessory PM
Case 3	3+	Myxomatous degeneration	62	AR, TR
Case 4	4+	Annular dilatation	52	PFO
Case 5	3+	A2 prolapse	74	–
Case 6	4+	A2 prolapse	62	AR

*MR* mitral regurgitation, *EF* ejection fraction, *ASD* atrial septal defect, *PFO* patent foramen ovale, *PM* papillary muscle, *AR* aortic valve regurgitation, *TR* tricuspid valve regurgitation

### Operative procedures

Five patients underwent MV plasty (MVP) and 1 patient received MV replacement (MVR). Anomalous formation of chordae tendineae was corrected by resection and suture with transplantation at the tip of the leaflet to which abnormal chordae were attached in 2 cases, while resection and suture with chordal shortening was performed in 1 case, and chordal reconstruction using artificial chordae (Gore-Tex, CV-4, W.L. Gore & Associates, Flagstaff, Ariz) was employed in 2 cases. For prolapse of the PML, the resection and suture technique (McGoon’s plication) was additionally performed in 4 cases, while the edge to edge fixation suture technique for commissural prolapse was performed in 2 cases.

Mitral-annuloplasty was subsequently carried out with the Physio-ring (Edwards Lifescience, Irvine, CA, USA) in 4 cases and the Saddle-ring (St. Jude Medical, St. Paul, MN, USA) in 1 case, with the average ring size of 29.2 ± 1.0 mm. A patient with combined valvular disease associated with supra-aortic valve stenosis initially received MVP with an aortic root enlargement procedure, but later underwent MVR, because residual MR was confirmed after cessation of aortic cross-clamping. As concomitant procedures, ASD closure in 2 cases, aortic valve replacement (AVR) in 2 cases (including 1 with combined aortic annular enlargement), and MAZE procedures in 1 case. The mean operation time was 308 ± 132 min, mean cardiopulmonary bypass time was 187 ± 87 min, and the mean aortic cross-clamp time was 125 ± 60 min. Operative data was shown in Table 3.

**Table 3:** Operative procedures

Patient no.	Operative procedures	Lesions	MAP (mm)
1	MVR 25 mm	–	–
2	Chordal reconstruction	A2	28
	Edge to edge fixation suture	PC	
3	Chordal translocation	A1	30
	Resection and suture	P1, P2	
	Edge to edge	AC	
4	MAP	–	28
5	Chordal translocation	A2	30
	Resection and suture	A2	
6	Chordal translocation + chordal shortening + resection and suture	A2	30

*MAP* mitral-annuloplasty, *MVR* mitral valve replacement

### Pathological findings

Histopathologic reports were obtained in 3 of 6 cases (pediatric group, 1; adult group, 2). The mitral leaflets with abnormal papillary muscle/chordae tendineae formation showed changes of myxomatous degeneration.

### Postoperative clinical course

There was no operative death. The average postoperative follow-up period was 7.8 ± 5.8 months (range, 1–20 months). Postoperative echocardiography showed no residual regurgitation in any of the cases and the status of all cases was declared as NYHA class I.

## Discussion

CMV dysplasia is a relatively rare and highly complex cardiac malformation that presents with abnormalities in the size and morphology of leaflets, chordae tendineae, and papillary muscles. Intra-cardiac anatomical anomalies, and a various degrees of morphological abnormalities such as anomalous formation of chordae tendineae, [2, 4, 5] and other congenital heart anomalies often coexist [6].

In the developmental process of MVs, chordae tendineae, and papillary muscle, a prominent horseshoe-shaped myocardial ridge appears from the anterior wall of the LV and extends to the posterior wall of the LV approximately on the 33rd fetal day, and this ridge connects with the endocardial cushion tissue in the atrio-ventricular region. Then, the myocardial ridge transforms into two papillary muscles and part of the endocardial cushion tissue differentiates into valve leaflets and chordae tendineae [7].

Oosthoek et al*.* studied human fetal hearts tissue with an electron microscope and hypothesized that in the process whereby the myocardial ridge of the LV wall connect with the endocardial cushion tissue at the atrio-ventricular septum and differentiate into papillary muscle and chordae tendineae at 5–19 fatal weeks, papillary muscle are found attached to valve leaflets without the involvement of chordae tendineae in cases of poor differentiation of endocardial cushion into chordae tendineae [1]. They also reported 28 cases of papillary muscle-chordae tendineae abnormalities in which one papillary muscle was developed from the high level of the LV and attached to AML directly or with slight mediation of chordae tendineae and called them asymmetric parachute-like mitral valves [2].

Echocardiography enables accurate evaluation of the morphology and function of valve leaflets, chordae tendineae, and papillary muscle. Carpentier and his colleagues performed operations on 47 pediatric patients with CMV dysplasia and classified the valvular lesions into 4 groups based on intraoperative findings: type I, mitral insufficiency due to failed MV coaptation with normal valvular movement; type II, mitral insufficiency due to prolapse with abnormal subvalvular tissue such as papillary muscle and chordae tendineae; type III, mitral stenosis and insufficiency; and type IV, mitral stenosis [8]. According to this classification, Case 1 was classified as type III, while Cases 2, 3, 5, 6 were type II and Case 4 as type I in our studies.

We propose that the reduced flexibility between the papillary muscles and MV leaflets due to the abnormality in papillary muscle/chordae tendineae was the likely mechanism of MR resulting in prolapse and tethering. Various surgical techniques for MVP have been reported and their mid- to long-term superiority to valve replacement has been reported [9]. The superiority of MVP in cases with CMV insufficiency has also been reported. Stellin et al. [10] performed MVP on 93 patients with CMV insufficiency spanning a period of 36 years and reported that early mortality was 7.5 % and late mortality was 8 %. They also confirmed that complex cardiac anomaly, mitral stenosis, and parachute MVs were risk factors for mortality [10]. Kudo et al. reported excellent outcomes of MVP with technique of the port access minimally invasive cardiac surgery for CMV insufficiency due to anomalous formation of papillary muscle [11].

In our experiences, MVP was feasible in five cases. Our strategies of MVP associated with undifferentiated papillary muscle are as follow; regurgitation resulted from the restriction of at the tip of the leaflet to which abnormal chordae attached was corrected by resection and suture with transplantation or chordal reconstruction using artificial chordae, and from the prolapse due to abnormal chordal elongation was corrected by means of chordal reconstruction or chordal shortening.

It was possible to reproduce an effective coaptation line and restore the flexibility of leaflets by transplantation or reconstruction of the chordae.

Although MVR has recently been carried out relatively safely, MVP is preferable unless complex congenital cardiac malformation coexisted, particularly in young patients, because it provides a wider valvular area with consideration of future physical growth and reduces the need for long-term anticoagulation treatment.

In a case of mitral insufficiency with supra-valvular aortic stenosis, initially MVP were performed using chordae transplantation and AVR with aortic annular enlargement was performed, however, TEE revealed residual MR after aortic clamping was released, and we finally performed MVR. One possible reason for residual MR was thought to be that an autologous pericardial patch interfered at the annular ring part of the AML and altered the geometry of the AML after MVP.

Papillary muscle/chordae tendineae abnormality is often accompanied by complex cardiac malformation, but no complication with congenital cardiac anomaly was present in the 3 adult patients and no symptoms were observed during a medical check-up. Since some cases with MR due to anomalous formation of papillary muscle and abnormal chordae tendineae showed an uneventful clinical course without symptoms, there may be potentially more cases of papillary muscle malformation than previously reported.

## Conclusions

MR associated with undifferentiated papillary muscle resulted from prolapse or tethering of MV leaflets at the attachment site and impaired flexibility of MV leaflets. It was possible to successfully treat the patients by MVP unless complex congenital cardiac malformation coexisted. Detailed examinations of MVs and attached papillary muscle by echocardiography and intraoperative inspection are necessary and surgical techniques should be selected appropriately in each case.
